# Fetal Cardiac Interventions—Are They Safe for the Mothers?

**DOI:** 10.3390/jcm10040851

**Published:** 2021-02-19

**Authors:** Beata Rebizant, Adam Koleśnik, Agnieszka Grzyb, Katarzyna Chaberek, Agnieszka Sękowska, Jacek Witwicki, Joanna Szymkiewicz-Dangel, Marzena Dębska

**Affiliations:** 1 2nd Department of Obstetrics and Gynecology, Centre of Postgraduate Medical Education, 01-809 Warsaw, Poland; chaberek.katarzyna@gmail.com (K.C.); drsekowska@gmail.com (A.S.); 2 Department of Perinatal Cardiology and Congenital Anomalies, Centre of Postgraduate Medical Education, US Clinic Agatowa, 03-680 Warsaw, Poland; akolesnik@cmkp.edu.pl (A.K.); agagrzyb@gmail.com (A.G.); jdangel@cmkp.edu.pl (J.S.-D.); 3 Cardiovascular Interventions Laboratory, The Children’s Memorial Health Institute, 04-730 Warsaw, Poland; 4 Department of Descriptive and Clinical Anatomy, Medical University of Warsaw, 02-004 Warsaw, Poland; 5 Department of Cardiology, The Children’s Memorial Health Institute, 04-730 Warsaw, Poland; 6 Pain Clinic, Department of Anesthesiology and Intensive Care, Centre of Postgraduate Medical Education, 00-416 Warsaw, Poland; 7 Department of Neonatology, Centre of Postgraduate Medical Education, 01-809 Warsaw, Poland; jacek.witwicki@bielanski.med.pl; 8 Department of Gynecologic Oncology and Obstetrics, Centre of Postgraduate Medical Education, 00-416 Warsaw, Poland

**Keywords:** fetal cardiac interventions, fetal interventions, fetal therapy, maternal safety, fetal valvuloplasty, stent placement, congenital heart disease, critical aortic stenosis, hypoplastic left heart syndrome, pulmonary atresia and intact ventricular septum

## Abstract

The aim of fetal cardiac interventions (FCI), as other prenatal therapeutic procedures, is to bring benefit to the fetus. However, the safety of the mother is of utmost importance. The objective of our study was to evaluate the impact of FCI on maternal condition, course of pregnancy, and delivery. 113 mothers underwent intrauterine treatment of their fetuses with critical heart defects. 128 percutaneous ultrasound-guided FCI were performed and analyzed. The patients were divided into four groups according to the type of FCI: balloon aortic valvuloplasty (fBAV), balloon pulmonary valvuloplasty (fBPV), interatrial stent placement (IAS), and balloon atrioseptoplasty (BAS). Various factors: maternal parameters, perioperative data, and pregnancy complications, were analyzed. There was only one major complication—procedure-related placental abruption (without need for blood products transfusion). There were no cases of: procedure-related preterm prelabor rupture of membranes (pPROM), chorioamnionitis, wound infection, and anesthesia associated complications. Tocolysis was only necessary only in two cases, and it was effective in both. None of the patients required intensive care unit admission. The procedure was effective in treating polyhydramnios associated with fetal heart failure in six out of nine cases. Deliveries occurred at term in 89%, 54% were vaginal. The results showed that FCI had a negligible impact on a further course of pregnancy and delivery.

## 1. Introduction

The majority of available data concerning fetal cardiac interventions (FCI) describe the effect of these procedures on the fetal outcome. Data concerning the influence of FCI on maternal condition are very limited.

The aim of fetal cardiac interventions is to change the natural course of some critical congenital heart defects (cCHD) [1,2,3,4,5] and, hence, to save fetal or neonatal life. Some CHD, if untreated prenatally, lead to irreversible changes in the anatomy and function of the cardiovascular system. In the case of severe aortic or pulmonary stenosis, the goal is to diminish the negative impact of pressure overload on the function and development of the left or right ventricle, respectively [3,6,7]. Opening the valve by ballooning unloads the ventricle and increases flow, allowing for better growth of the ventricle and vessels, according to the “no flow—no growth” theory [8]. In the cases of left heart disease with closed foramen ovale, the aim of the intervention is to prevent the damage of pulmonary vessels that would inevitably lead to life-threatening pulmonary hypertension and often irreversible anatomical changes in pulmonary circulation [5,9,10,11,12]. However, in such cases, FCI also aims to improve the maternal condition, as these defects commonly cause severe polyhydramnios, which may significantly affect maternal health and the course of pregnancy.

An increasing number of international data suggest that FCI enables the improvement of fetal condition [13,14]. This is a unique situation in which the fetus requires treatment, but the healthy mother is exposed to the risk of potential complications. Therefore, ensuring the woman’s safety is very important. The aim of our study is to assess the impact of FCI on maternal condition, as well as the further course of pregnancy and delivery.

## 2. Materials and Methods

### 2.1. General Study Design

Between June 2011 and April 2020, our team performed 128 percutaneous ultrasound-guided fetal cardiac interventions on 113 fetuses [15]. This research included all of the patients undergoing FCI in the 2nd Department of Obstetrics and Gynecology of the Centre of Postgraduate Medical Education in Bielanski Hospital, Warsaw, Poland. Prospective and retrospective analyses of medical records and patients’ charts were performed. The Ethical Committee of the Centre of Postgraduate Medical Education approved the study. The mothers signed an informed consent form and an agreement for the experimental therapy.

Prior to hospital admission, all of the FCI candidates had undergone fetal echocardiography at the Department of Perinatal Cardiology and Congenital Anomalies, Centre of Postgraduate Medical Education, US Clinic Agatowa, Warsaw, Poland.

### 2.2. Patients/Study Population

The patients were qualified for the intervention based on published inclusion criteria [6,16,17,18] by experienced pediatric cardiology and maternal-fetal medicine specialists.

The maternal exclusion criteria were, as follows: any diseases that increment the maternal risk of either the procedure or anesthesia or impaired psychological condition.

### 2.3. Management

Following a detailed individual evaluation, parents were offered three possibilities: (1) close prenatal follow-up, without FCI with optimal timing of delivery; (2) fetal cardiac intervention; and (3) the termination of pregnancy. All the patients but three opted for an attempt of prenatal treatment. One mother decided to terminate the pregnancy due to a poor prognosis, and two others resigned after giving initial consent.

All of the FCI were percutaneous ultrasound-guided procedures. Three different ultrasound machines were used: Accuvix A30, probes: C2-61C, 2–6 MHz; Samsung WS80A, probes: CV1-8A, CA1-7A; and Epiq 7, probe C9-2. Ultrasound recordings were collected on DVDs or external drives.

Mothers’ preoperative preparation was conducted according to the local protocol: eight hours preoperative fasting period was required, ranitidine and metoclopramide for prophylaxis of aspiration pneumonia [19,20], intravenous hydration, and antibiotic prophylaxis 30 min. before the intervention were administered.

### 2.4. Anesthesia

Eleven FCI were performed under general anesthesia, and 117 procedures were performed in conscious analgosedation of the mother with fentanyl and midazolam. After choosing the cardiac puncture site, we additionally performed maternal local anesthesia by injecting lidocaine along the planned needle trajectory from the skin to the uterine wall. The fetus was separately anesthetized with fentanyl (20 mcg/kg EFW—estimated fetal weight), and atracurium (0.2 mg/kg EFW) administered into the umbilical vein [21,22,23].

### 2.5. Cordocentesis and Cardiac Puncture

All of the cordocenteses were performed with a 22-gauge needle (Spinocan, B. Braun Melsungen AG, Melsungen, Germany). For cardiac interventions, we used needles in two sizes: 18-gauge (Cook Medical Trocar Needle; Cook Medical Inc., Bloomington, IN, USA) and 17-gauge (Argon Medical Devices, Co-Axial Introducer needle, Argon Medical Devices Inc., Athens, TX, USA), depending on the balloon diameter. A 17-gauge needle was used for a balloon sized 4.5 mm or more. For pericardial drainage, a 20-gauge needle or a 21-gauge needle was used (Cook Medical Echotip Disposable Amniocentesis Needle; Cook Medical Inc., Bloomington, IN, USA). The technical details of the procedures were described in our previous papers [15,24]. In most cases, the whole procedure was performed with only two or three needle punctures (cordocentesis, cardiac puncture, and pericardial drainage or drug administration).

### 2.6. Transplacental Fetal Treatment

In order to improve fetal cardiac function, we introduced transplacental treatment with digoxin, using its positive inotropic effect. Before drug initiation, maternal contraindications for the therapy were excluded, a maternal electrocardiogram was performed, and serum potassium concentration was measured. A loading dose of 0.5 mg given three times a day was administered to the pregnant woman for the first two days. Afterward, the maintenance dose of 0.25 mg three times a day was continued. For maternal safety, we monitored the digoxin and potassium level in maternal serum, observed the clinical symptoms of digoxin overdosage, and performed electrocardiogram (ECG) monthly.

### 2.7. Data Collection

A database was created to collect the following parameters: maternal age, paternal age, maternal body mass index (BMI), family history of CHD, gestational age at intervention, gestational age at delivery, mode of delivery, number of uterus punctures, ASA (American Society of Anesthesiologists) physical status classification, method of anesthesia, and anesthesia-associated complications. Postoperative conditions, including nausea and vomiting requiring pharmacological intervention, postoperative pain demanding analgetic treatment, aspiration, the need for blood products transfusion within seven days after delivery, tocolysis administration, preterm birth, preterm prelabor rupture of membranes (pPROM), intrauterine infection (defined as the presence of the following findings: temperature >38 °C, uterine tenderness, maternal tachycardia >100/min., fetal tachycardia >160/min [25]), the administration of digoxin, digoxin-related ailments, intensive care unit admission, and maternal death were also analyzed.

Patients were divided into four groups according to the type of fetal cardiac intervention: balloon aortic valvuloplasty (fBAV), balloon pulmonary valvuloplasty (fBPV), interatrial stent placement (IAS), and balloon atrioseptoplasty (BAS).

### 2.8. Statistical Analysis

Statistical analyses were performed with Statistica 13.3 (Tibco Software Inc., Palo Alto, CA, USA). The data were expressed as mean ± SD or median with range, when appropriate. If there was a statistically significant difference between any two of the groups, *t*-student or non-parametric test was used, depending on the variable distribution. Differences between more than two groups were calculated with Kruskal-Wallis H test followed by a multiple comparison post-hoc test. Pearson and rho-Spearman correlations, and Fisher’s exact test were also used, when appropriate. A *p*-value < 0.05 was considered to be statistically significant.

## 3. Results

### 3.1. Number and Types of FCI

**Figure 1:**
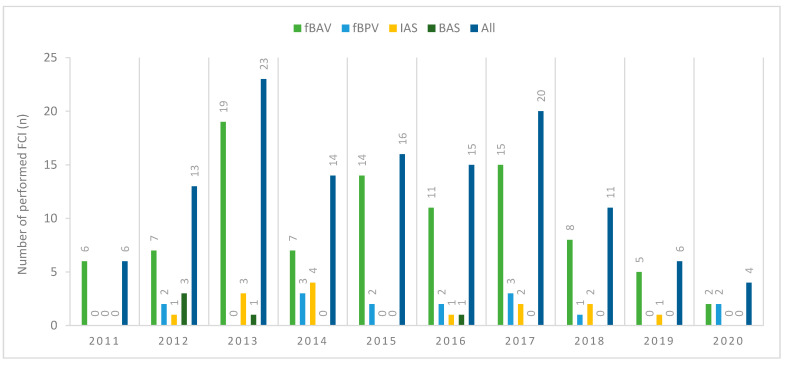
Number of fetal cardiac interventions (FCI) performed per year (from June 2011 until April 2020). fBAV: fetal balloon aortic valvuloplasty; fBPV: fetal balloon pulmonary valvuloplasty; IAS: interatrial stent placement; BAS: balloon atrial septoplasty; FCI: fetal cardiac intervention.

Between June 2011 and April 2020, 128 fetal cardiac interventions were performed in 113 patients: 94 aortic balloon valvuloplasties in 88 patients; 15 pulmonary balloon valvuloplasties in 13 patients; 14 interatrial stent placements in 14 patients; and, five balloon atrial septoplasties in five patients. Figure 1 presents the number of fetal cardiac interventions performed yearly.

In eight mothers, the same procedure was performed twice due to either technical failure of the first FCI or disease progression, five patients had two different procedures, and one patient had three different procedures.

### 3.2. Characteristics of the Patients

112 patients (99%) were Polish citizens, and all of them were Caucasian. One patient, referred to our center from Vietnam, was an American citizen. The median maternal age at FCI was 30 years (range 18–41), median paternal age was 32 years (range 20–54), with no significant differences between groups of procedures.

Before the procedure, the patients were consulted by an anesthesiologist. All of them were considered to be in good health, but as pregnant were automatically classified as ASA 2 [26].

The median BMI (body mass index) of the mothers was 24.6 (range 17.8–38.7); there was no difference in maternal BMI between the groups. Median gestational age at the first procedure was 25 weeks of gestation (range 20–32 weeks). Four women (3.5%) were active smokers during pregnancy and, in two of these pregnancies, intrauterine fetal demise classified as nonrelated to the procedure occurred.

Table 1 presents characteristics of the patients who underwent FCI according to the type of intervention.

### 3.3. Technical Aspects of FCI

All 128 procedures were performed under continuous ultrasound guidance with the percutaneous transabdominal approach. The placental location was posterior in 60% and anterior in 40% of cases. 115 procedures (90%) were performed with an 18-gauge needle. During 13 procedures (10%), a 17-gauge needle was used due to balloon diameter 4.5 mm or more. The median number of uterus needle punctures necessary to complete the procedure was 3 (range 2–11, no significant differences between the types of intervention) and 98 out of 128 procedures (77%) were completed with no more than three punctures. More uterine punctures per procedure were observed in patients with posterior placenta (median 3, range 2–11 vs. median 3, range 2–5, *p* = 0.016). In those cases, we more easily decided to perform the intervention in cases with a suboptimal fetal position, which required additional needle maneuvers. Fetal cardiac tamponade decompression was necessary during 84 interventions (65%). Eleven punctures were needed in one case of successful fetal resuscitation following a balloon rupture within the pulmonary artery.

### 3.4. Threatened Premature Labor

Tocolysis was not routinely administered—neither before, nor during, nor after the procedures. Only two patients developed transient preterm contractions following FCI. In both of them, short term tocolysis (48 h) with betamimetics was successful. Finally, one of the patients delivered 69 days following the procedure (at 36 weeks and three days), and the second had an elective cesarean section in a different center, 41 days following the procedure (at 34 weeks of gestation). There were no preterm births due to the preterm uterine contractions that occurred within 10 days post-FCI. One patient (primigravida, 28 weeks of gestation) needed rescue cervical cerclage prior to the fetal cardiac intervention, due to preexistent cervical insufficiency (cervical length 11 mm). The FCI was safely performed two days after successful cerclage.

### 3.5. pPROM

None of the 128 procedures was complicated with procedure-related pPROM (defined as pPROM within 10 days following the intervention). There were nine cases of pre-existing polyhydramnios that are caused by fetal cardiac failure; six of them resolved following a successful FCI. In the remaining three cases, polyhydramnios persisted and in 2 of them resulted in later pPROM and preterm birth, which was nonrelated to the procedure.

### 3.6. Postoperative Events

No significant maternal bleeding occurred. There was one case of placental abruption that was related to the procedure, but the mother did not need any blood products transfusion. None of the mothers required intensive care unit admission. Postoperative nausea and vomiting on the day of intervention were reported by three patients—all of which had undergone general anesthesia with intubation. There were no other anesthesia-associated complications. All of the patients found anesthesia effective.

Eight patients reported mild abdominal pain post-FCI in the area of the punctures. It was usually observed in patients who required more needle punctures during the procedure (median 4.5, range 3–11 vs. median 3, range 2–6, *p* = 0.001, *r* = 0.32). In all cases, the pain was sufficiently controlled with acetaminophen. None of the patients needed opioids administration following FCI.

Table 2 presents procedure information, maternal morbidity, and mortality.

### 3.7. Digoxin Transplacental Therapy

Ninety-two patients received digoxin postoperatively to improve fetal cardiac function. In 31 cases, the maintenance dose (0.25 mg three times a day) was reduced due to side effects—mostly mild nausea and vomiting. Three mothers required hospital admission due to intensive nausea and vomiting while taking the drug. Only in one of them, digoxin serum concentration exceeded the therapeutic level 1.5–2.5 ng/mL [27], and it was 2.51 ng/mL. In the other two cases, adverse effects occurred when the drug level was 1.55 ng/mL and 1.96 ng/mL, respectively, and resolved soon after dose reduction.

All mothers who received digoxin had characteristic ECG changes (downsloping ST-segment depression). None of them had severe ECG changes, which could be dangerous for them. There were no cases of digoxin therapy cessation due to abnormal ECG.

### 3.8. Pregnancy Outcomes

#### 3.8.1. All Patients

Median gestational age at delivery was 39 weeks (range 29–41). There were 100 live births (out of 113 pregnancies)—89 term deliveries (89%), and 11 preterm deliveries (11%). Only six preterm deliveries occurred before 34 weeks of gestation.

The general rate of cesarean section was 46%, and the value is similar to the general cesarean section rate in our country. The majority of operative deliveries was performed due to obstetric indications. In two cases, cesarean sections were performed to optimize the logistic conditions of the postnatal care.

There were no terminations of pregnancy in the treated group. Intrauterine fetal demise related to the procedure occurred in nine cases. There was one procedure-related postnatal death following preterm delivery in the 25th week of gestation. In that case, placental abruption occurred within 48 h following the intervention. This complication possibly resulted from extensive needle maneuvers being performed through the anterior placenta to correct the fetal position. After that case, we avoided such maneuvers if the placenta was anterior. There were three cases of intrauterine fetal demise nonrelated to the procedure; in two of these cases, mothers were heavy smokers.

Table 3 presents data regarding pregnancy.

#### 3.8.2. Patients Who Underwent More than One FCI

Fourteen mothers underwent more than one procedure. In this group median gestational age at delivery was also 39 weeks (range 29–40). There were 13 live births (93%)—10 term deliveries (77%) and three preterm deliveries (23%). Only one preterm delivery occurred before 34 weeks of gestation. It was a pregnancy complicated with severe cardiac failure and polyhydramnios, where polyhydramnios persisted despite three different FCI and two amnioreductions. There was one case of procedure-related death in this group (7%).

## 4. Discussion

Discussing the potential maternal risk of intrauterine intervention with the pregnant woman should be an inherent part of the consultation preceding every interventional fetal therapy. Intrauterine fetal procedures create exceptional conditions, in which at least, from the medical point of view, the mother does not benefit, but can suffer from potential complications. It is essential to minimalize maternal risk, which should never outweigh the possible improvement of the fetal condition. When running the FCI program at our center for the past nine years, we made an effort to improve not only the technical aspects of the procedures performed on the fetus, but also to ensure maternal safety.

### 4.1. Anesthesia

The first issue to discuss is the anesthesia for fetal therapy, which should ensure either safety or comfort for both the mother and the fetus [22,28]. The majority of FCI worldwide used to be performed under maternal general anesthesia with intubation, according to the published data [3,4,5,29,30]. However, this trend has been changing in recent years.

It is well known that pregnant women are at an increased risk of difficult intubation and aspiration of digestive contents into the respiratory tract [31,32,33]. Therefore, healthy pregnant women are routinely considered by anesthesiologists as ASA 2 [26]. Failed intubation is the most common cause of anesthesia-related maternal death [34].

The initial eight FCI procedures were performed under general anesthesia with intubation, according to the literature available at that time. Each time we separately performed cordocentesis for either fetal blood sampling (to assess fetal karyotype and blood parameters) or separate anesthesia of the fetus (with fentanyl and atracurium).

Based on those procedures, we realized that, with the same route and number of needle punctures, we can provide efficient fetal anesthesia. Therefore, we moved from general anesthesia to the mother’s conscious analgosedation. Moreover, before introducing the 18 or 17-gauge needle, we additionally injected lidocaine locally along the planned needle trajectory. Conscious maternal analgosedation, combined with local anesthesia of the puncture site, turned out to be sufficient for maternal anesthesia. The fetus was successfully anaesthetized separately with fentanyl and atracurium administered into the umbilical vein.

Henceforth, the mother’s general anesthesia with intubation was only necessary in three cases due to FCI complications that prolonged the procedure and increased the number of uterine punctures.

Additionally, such anesthesia can be also offered to patients with high-risk intubation, e.g., patients with goiter, obesity, or other anatomical factors increasing the difficulty of intubation. Other benefits of conscious analgosedation of the mother include smaller doses of anesthetic drugs administered, the greater stability of her circulatory system, and more stable uteroplacental flow [20,35,36]. Following such anesthesia, patients were more likely to experience greater comfort, fewer adverse effects, and faster return to normal activity. In our group, we observed significantly less nausea and vomiting associated with anesthesia than reported by other authors [29].

### 4.2. Approach

All of the fetal cardiac interventions were performed through the needle inserted into the mother’s abdomen under ultrasound guidance, which was consistent with most other reports [29,37,38,39], but it was not a uniformly applied route. The literature contains reports of performing laparotomy to optimize fetal position for needle procedure and enhance imaging resolution when the ultrasound probe was placed directly on the uterine wall [4,30,40]. This approach was used by some centers in the past, but it increased the risk of maternal complications, so it was abandoned.

In some cases, if the fetal position was not perfect, but close to optimal, it was possible to change it with the needle. In cases of an unfavorable fetal position, we first applied external maneuvers. Eventually, if fetal position was close to optimal, we corrected it with a 18/17-gauge needle that was inserted into the uterus for the cardiac procedure. If the position of the fetus was far from optimal, we waited for its spontaneous change asking mothers to take a walk or do same exercises. If this was unsuccessful, we postponed the intervention. Our experience shows that extensive maneuvers with the needle across the anterior placenta should be avoided in order to prevent placental bleeding or detachment.

### 4.3. Uterine Punctures

As mentioned before, the majority of procedures (77%) only required two or three uterine punctures. In our opinion, such a minimally invasive approach resulted in the elimination of the need for tocolysis and prevented procedure-related pPROM, which was not observed in any of our patients.

In other types of prenatal therapy, the rate of maternal complications is higher [37]. According to the literature, the rate of pPROM after fetoscopy and open fetal surgery exceeds 30% [41,42,43]. Some of the authors provide data that the overall rate of maternal complications after fetoscopy (such as chorioamnionitis, placental abruption, ICU-admission, and others) reaches 17.4% [41].

### 4.4. Digoxin

Our patients received postoperatively relatively high doses of digoxin, which could raise safety-related concern. The chosen dosing was based on the literature review [27,44,45] and it has its background on given in the literature rate of the drug passing through the placenta to the fetus (feto-maternal ratio). Our results in this field will be addressed in another paper [46]. Nevertheless, complications that were related to digoxin toxicity were rare and mild.

Interestingly, the recommended drug serum level (1.5 ng/mL–2.5 ng/mL) was slightly exceeded in only one of the patients reporting side effects [27].

### 4.5. The Course of Pregnancy and Delivery

Our experience suggests that the influence of fetal cardiac interventions on pregnancy and delivery was negligible. In cases of successful fetal intervention, further course of pregnancy was uneventful and the performed intervention itself did not influence the time and mode of delivery. Moreover, as in most cases, polyhydramnios resulting from cardiac failure, resolved after the procedure, the FCI probably prevented pregnancy complications, like cervical shortening, preterm delivery, and mirror syndrome.

Neither FCI nor the fetal heart defect were indications for a cesarean section.

### 4.6. Limitations of the Study

The limitations of the study mainly concerned the groups that were covered by the study. Firstly, it was an observational single-center analysis, and the number of patients was relatively limited. Secondly the heterogeneity of the critical congenital heart diseases, the differences in patient selection criteria used between FCI centers and a variability of individual pregnancy course make comparing the outcomes difficult. However, despite all these limitations, intrauterine cardiac procedures could be offered, as very promising interventions to selected cases, taking the fact that these are relatively safe procedures to the mothers into account.

## 5. Conclusions

Maternal complications of any fetal intervention usually reflect the degree of its invasiveness (fetal approach, the type and size of devices used, and number of uterine punctures).

Fetal cardiac interventions are needle procedures are performed with relatively thin needle being introduced through the maternal abdominal layers under ultrasound guidance. If performed efficiently with minimum invasiveness by experienced operators in possibly safe and effective anesthesia, FCI are one of the safest for the mother’s intrauterine procedures in modern prenatal therapy.

Conscious maternal analgosedation with separate fetal anesthesia can be successfully used in most FCI, avoiding complications of general anesthesia and not increasing the procedure-related pain.

In cases with pre-existing risk of preterm delivery (polyhydramnios, short cervix), the decision about the intervention should be made on an individual basis, weighting the risk of prematurity against the risk of fetal cardiovascular deterioration.

Digoxin dosage should be adjusted based on both clinical observations (maternal side effects, ECG changes) and the serum drug level, because there is no strong correlation between them.

## Figures and Tables

**Table 1:** Characteristics of the patients who underwent fetal FCI according to the type of intervention.

Characteristic	fBAV (*n* = 88)	fBPV (*n* = 13)	IAS (*n* = 14)	BAS (*n* = 5)	All (*n* = 113)	*p*-Value
Maternal age at FCI (years)	30 (18–41)	33 (26–39)	29 (22–35)	27 (25–34)	30 (18–41)	0.070
BMI (kg/m^2^)	24.7 (17.8–38.7)	24.1 (19.3–29.7)	24.4 (18.8–30.1)	24.6 (20.8–28.1)	24.6 (17.8–38.7)	0.650
Obesity (BMI > 30 kg/m^2^)	14 (15.9%)	0 (0%)	1 (7.1%)	0 (0%)	15 (13.3%)	0.279
Gravidity	2 (1–5)	2 (1–5)	2 (1–3)	2 (2–5)	2 (1–5)	0.433
Parity	1 (0–3)	0 (0–2)	1 (0–2)	2 (1–2)	1 (0–3)	0.091
Active smoker	4 (4.5%)	0 (0%)	0 (0%)	0 (0%)	4 (3.5%)	0.684
Paternal age (years)	32 (20–46)	34 (26–54)	32 (24–36)	34 (26–39)	32 (20–54)	0.702
Family history of CHD	8 (9.1%)	0 (0%)	0 (0%)	0 (0%)	8 (7.1%)	0.378
ASA physical status	ASA 2–100%	ASA 2–100%	ASA 2–100%	ASA 2–100%	ASA 2–100%	1.000

Data are given as median (range) or *n* (%). *p*-value was calculated with the Kruskal-Wallis test. fBAV: fetal balloon aortic valvuloplasty; fBPV: fetal balloon pulmonary valvuloplasty; IAS: interatrial stent placement; BAS: balloon atrial septoplasty; FCI: fetal cardiac intervention; BMI: body mass index; CHD: congenital heart defect; ASA: American Society of Anesthesiologists.

**Table 2:** Perioperative data, maternal morbidity, and mortality for all 128 FCI procedures according to the type of intervention.

	fBAV (*n* = 94)	fBPV (*n* = 15)	IAS (*n* = 14)	BAS (*n* = 5)	Total (*n* = 128)	*p*-Value
Total number of uterus punctures (cordocentesis, cardiac puncture, decompression of tamponade, drug administration)	3 (2–5)	3 (2–11)	3 (2–8)	3 (2–4)	3 (2–11)	0.103
Tocolysis	2 (2.1%)	0 (0%)	0 (0%)	0 (0%)	2 (1.6%)	0.866
Procedure-related pPROM ^&^	0 (0%)	0 (0%)	0 (0%)	0 (0%)	0 (0%)	1.000
Procedure-related placental abruption ^$^	1 (1.1%)	0 (0%)	0 (0%)	0 (0%)	1 (0.8%)	0.948
Postoperative nausea and vomiting	2 (2.1%)	0 (0%)	0 (0%)	1 (20%)	3 (2.3%)	0.058
Postoperative pain	3 (3.2%) ^#^	3 (20%) ^#^	1 (7.1%)	1 (20%)	8 (6.3%)	0.048
Bleeding requiring blood transfusion	0 (0%)	0 (0%)	0 (0%)	0 (0%)	0 (0%)	1.000
Chorioamnionitis	0 (0%)	0 (0%)	0 (0%)	0 (0%)	0 (0%)	1.000
Wound infection	0 (0%)	0 (0%)	0 (0%)	0 (0%)	0 (0%)	1.000
Post-FCI hospitalization (days)	4 (1–84)	5 (1–7)	5 (2–70)	3 (2–19)	4 (1–84)	0.412
Intensive care unit admission	0 (0%)	0 (0%)	0 (0%)	0 (0%)	0 (0%)	1.000
Intra-/perioperative maternal mortality	0 (0%)	0 (0%)	0 (0%)	0 (0%)	0 (0%)	1.000

Data are given as median (range) or *n* (%). *p*-value was calculated with Kruskal-Wallis test and multiple comparisons post-hoc test. fBAV: fetal balloon aortic valvuloplasty; fBPV: fetal balloon pulmonary valvuloplasty; IAS: interatrial stent placement; BAS: balloon atrial septoplasty; ^&^ pPROM within 10 days from FCI; ^$^ placental abruption within 10 days from FCI; ^#^ *p* = 0.009.

**Table 3:** Pregnancy outcomes according to the type of fetal cardiac intervention.

	fBAV	fBPV	IAS	BAS	Total	*p*-Value
Number of procedures/number of fetuses	94/88	15/13	14/14	5/5	128/113	NA
Gestational age at FCI (weeks)	25 (20–32)	24 (22–30)	27 (22–33)	24 (22–29)	25 (20–33)	0.407
Procedure-related death of the fetus/neonate ^†^	7	1	2	1	10	0.655
Procedure non-related death of the fetus	3	0	0	0	3	0.776
Live birth	78	12	12	4	100	0.889
Gestational age at birth (weeks)	39 (29–41) ^§^	38 (33–40)	37 (29–41)	33.5 (29–39) ^§^	39 (29–41)	0.041
Mode of delivery VD/CS	42/36 54%/46%	6/6 50%/50%	7/5 58%/42%	3/1 75%/25%	54/46 54%/46%	0.837
Time interval between intervention and birth (days)	92 (17–162)	100 (46–117)	75 (11–109)	57.5 (17–114)	92 (11–162)	0.025
Birth weight (g)	3330 (950–4950) ^#^	3130 (2100–3920) ^^^	3130 (1770–4090)	2640 (1800–2860) ^#,^^	3320 (950–4950)	0.046

Data are given as median (range), number or %. *p*-value calculated with the Kruskal-Wallis test and multiple comparisons post-hoc test. CS: cesarean section; VD: vaginal delivery; NA: not applicable; ^†^ procedure-related death—fetal/neonatal death within 72 h following the procedure; ^§^ *p* = 0.030 (fBAV vs. BAS); ^#^ *p* = 0.001 (fBAV vs. BAS); ^^^ *p* = 0.048 (fBPV vs. BAS). Column “Total” is not the sum of previous columns, because in 14 patients more than one procedure was performed (the same FCI twice in eight cases, two different FCI in five cases, and three different FCI in one case).

## Data Availability

The data presented in this study are available on request from the corresponding authors. The data are not publicly available due to privacy regulation.
